# T2DM Self-Management via Smartphone Applications: A Systematic Review and Meta-Analysis

**DOI:** 10.1371/journal.pone.0166718

**Published:** 2016-11-18

**Authors:** Mingxuan Cui, Xueyan Wu, Jiangfeng Mao, Xi Wang, Min Nie

**Affiliations:** Department of Endocrinology, Peking Union Medical College Hospital, Peking Union Medical College, Chinese Academy of Medical Sciences, Beijing, China; Key laboratory of Endocrine, National Health and Family Planning Commission, Beijing, China; Florida International University Herbert Wertheim College of Medicine, UNITED STATES

## Abstract

**Background:**

Mobile health interventions (mHealth) based on smartphone applications (apps) are promising tools to help improve diabetes care and self-management; however, more evidence on the efficacy of mHealth in diabetes care is needed. The objective of this study was to conduct a systematic review and meta-analysis of randomized controlled trials (RCTs) assessing the effect of mHealth apps on changes in hemoglobin A1c (HbA1c), blood glucose, blood pressure, serum lipids, and body weight in type 2 diabetes mellitus (T2DM) patients.

**Methods:**

Two independent reviewers searched three online databases (PubMed, the Cochrane Library, and EMBASE) to identify relevant studies published between January 2005 and June 2016. Of the 2,596 articles retrieved, 13 RCTs were included. We used random effects model to estimate the pooled results.

**Results:**

Thirteen studies were selected for the systematic review, six of which with data available containing 1,022 patients were included for the meta-analysis. There was a moderate effect on glycemic control after the mHealth app-based interventions. The overall effect on HbA1c shown as mean difference (MD) was -0.40% (-4.37 mmol/mol) (95% confidence interval [CI] -0.69 to -0.11% [-7.54 to -1.20 mmol/mol]; p = 0.007) and standardized mean differences (SMD) was -0.40% (-4.37 mmol/mol) (95% confidence interval [CI] -0.69 to -0.10% [-7.54 to -1.09 mmol/mol]; p = 0.008). A subgroup analysis showed a similar effect with -0.33% (-3.61 mmol/mol) (95% CI -0.59 to -0.06% [-6.45 to -0.66 mmol/mol]; p = 0.02) in MD and -0.38% (-4.15 mmol/mol) (95% CI -0.71 to -0.05% [-7.76 to -0.55 mmol/mol]; p = 0.02) in SMD in studies where patients’ baseline HbA1c levels were less than 8.0%. No effects of mHealth app interventions were found on blood pressure, serum lipids, or weight. Assessment of overall study quality and publication bias demonstrated a low risk of bias among the six studies.

**Conclusions:**

Smartphone apps offered moderate benefits for T2DM self-management. However, more research with valid study designs and longer follow-up is needed to evaluate the impact of mHealth apps for diabetes care and self-management.

## Introduction

Diabetes mellitus is one of the most common chronic diseases affecting humans and is rapidly expanding in prevalence worldwide[1]. To a great extent, unhealthy lifestyle contributes to T2DM [2] and one of the mainstays of treatment and prevention of diabetes is adopting a healthy lifestyle [3]. As there is no cure for diabetes, self-management plays a vital role over the course of one’s lifetime. Self-management includes measuring and recording blood glucose, exercising, maintaining a diabetes diet, and regularly taking medication. Many studies have indicated that improvement in dietary and physical activity habits can prolong life expectancy in patients with T2DM [4, 5].

Self-management tools are developing fast. Data recording of blood glucose and life-style changes has progressed from recording them in paper to uploading them to computers and using traditional phone functions, and finally to using smartphone apps. Many systematic reviews and meta-analyses proved that mHealth tools are effective in self-management in a wide range of areas both for disease-management and life-style changes in daily life [6–8]. Previous reviews of the mHealth tools have focused largely on text messaging, phone calls, or computer-based or laptop/tablet-based interventions rather than focusing on smartphone apps[9].

Some recent studies have shown that among the diverse computer-based technologies, mobile phone interventions for diabetes self-management have been able to significantly reduce HbA1c levels; this may be related to feedback or interaction between patients and providers in the implementation process as well as the advantages of mobile phones such as adherence, intensity of the interventions and the behavior-change techniques used by the interventions [10, 11]. Nowadays, smartphone users are increasing rapidly employing diverse apps to help with T2DM self-management[12]. The function of these apps aims mainly at monitoring clinical values, such as HbA1c, blood glucose, blood pressure, serum lipids, and body weight uploaded by users. As a new type of management model, mHealth interventions offer T2DM patients a way to overcome the shortcoming of traditional health tracking methods by providing convenience and medical care in daily life and minimizing the distance, time, and cost. However, more evidence is still needed regarding the effectiveness of these apps. Thus far, results among different studies have been inconsistent[13, 14].

The objective of this study was to conduct a systematic review and meta-analysis of randomized controlled trials (RCTs) assessing the impact of mobile phone apps with or without feedback compared to usual care alone on the management of adult patients with T2DM. Values of interest included changes in HbA1c, blood glucose, blood pressure, low-density lipoprotein cholesterol (LDL-c), high-density lipoprotein cholesterol (HDL-c), triglyceride (TG), total cholesterol (TC), and body weight.

## Methods

This systematic review and meta-analysis was conducted following the Preferred Reporting Items for Systematic Review and Meta-Analyses (PRISMA) statement[15].

### Data sources and searches

A literature search with no language restriction was performed using PubMed, the Cochrane Library, and EMBASE databases to identify relevant studies published from 2005 through June 2016. Combinations of the following MESH terms and keywords were used to search the different databases: *type 2 diabetes mellitus*, *glycemic control*, *self-management*, *smartphone*, *and mobile applications*. For detailed search strategies please see S1 Table.

### Study selection

The studies selected were RCTs that compared mHealth application interventions in T2DM self-care in patients ≥18 years old with the control group using usual care only. We restricted our inclusion criteria to trials that evaluated at least one of the primary or secondary outcomes mentioned below. The primary outcome was change in HbA1c values. Secondary outcomes were changes in blood pressure, serum lipids, weight, and life-style behaviors. Selected studies of type 2 diabetes were not restricted to patients of any particular race.

Exclusion criteria included: (1) gestational diabetes mellitus; (2) type 1 diabetes mellitus; (3) systematic review and meta-analysis; (4) mobile-based interventions via phone calls or short message service; (5) studies with less than 3 months of follow-up; and (6) duplicate publications or sub-studies of included trials.

### Data extraction and quality assessment

Two authors independently reviewed the abstracts of studies retrieved from the database search and read the full text of potentially-relevant articles. For studies that met the inclusion criteria, data extraction was independently conducted by two investigators using standard data extraction templates. Disagreements in data extraction were solved by a third investigator. Quality of the included studies was assessed by using the Cochrane Collaboration’s tool, which evaluates random sequence generation, allocation concealment, blinding of participants and personnel, blinding of outcome, incomplete outcome data, selective reporting, and other sources of bias. The Cochrane Collaboration’s Review Manager software was used to perform a meta-analysis that pooled the mean changes related to the selected outcomes from the data sources. For studies in which outcome data were not suitable for meta-analysis, the information was described in phrases (Table 1).

**Table 1:** Informations of selected studies.

Author, year	Number of subjects/Age (years) mean (SD)	Types ofstudy/duration	country (setting)	Type of apps and Methodology	Feedback	Summary of results
Karhula T, 2015[16]	Intervention group: n = 180/66.6(8.2) Control group: n = 70/65.5(9.6)	Randomized controlled trials/1 year	Finland (Mobile phones)	A remote patient monitoring (RPM) system (a mobile phone with specific software,a blood pressure meter). The intervention group: Using the RPM system via mobile phones to self-monitor weight, blood pressure and blood glucose once per week.	A personal health coach called the patients once every 4 to 6 weeks.	The intervention group: A significant decrease in weight, waist circumference, SBP, DBP, and LDL-cholesterol. The control group: A significant decrease in SBP and LDL-cholesterol.
Holmen H, 2014[17]	FTA: n = 51/58.6(11.8) FTA-HC: n = 50/57.4(12.1) Control group: n = 50/55.9(12.2)	Randomized controlled trials/1 year	Norway (Mobile phones)	A mobile phone–based self-management system: Few Touch Application (FTA) (A blood glucose-measuring system with a diabetes diary app on mobile phone). Two intervention groups: FTA group and FTA-HC (health coaching) group.	Health counseling for the first 4 months in FTA-HC group.	All three groups: HbA1c level decreased but not significantly different. FTA-HC group: The heiQ (health-related quality of life) domain skills and technique acquisition was significantly greater.
Orsama AL, 2013[18]	Intervention group: n = 24/62.3(6.5) Control group: n = 24/61.5(9.1)	Randomized controlled trials/10 months	Finland (Mobile phones)	A mobile phone with an application "Monica" to report the health parameters (blood pressure, weight, physical activity, and blood glucose values). The intervention group: Providing technology (mobile telephone, software application and assessment devices) to monitor diabetes health-related parameters	Graphs and feedback messages were sent to patients after each upload. Study nurses scanned the data each week and contacted patients if necessary.	A mean reduction in HbA1c of -0.40% (95% [CI] -0.67% to -0.14%) versus 0.036% (95% CI -0.23% to 0.30%)(P < 0.03) and weight reduction of -2.1 kg (95% CI -3.6 to -0.6 kg) versus 0.4 kg (95% CI -1.1 to 1.9 kg)between intervention and control group.
Quinn CC, 2011[19]	Group 1: UC n = 56/53.2(8.4) Group 2: CO n = 23/52.8(8.0) Group 3: CPP n = 22/53.7(8.2) Group 4: CPDS n = 62/52(8.0)	Randomized controlled trials/1 year	U.S. (Mobile phones)	A patient-coaching system: a mobile software application(to enter diabetes self-care data:blood glucose values, carbohydrate intake and medications) and a web portal. The intervention group: Providing a mobile phone with app to receive real-time educational messages specific to the entered data.	The feedbackalgorithm sent educational and motivational messages to patients after each data upload.	The intervention group: The mean declines in HbA1c were 1.9%. The control group: The mean declines in HbA1c were 0.7%.(D (P = 0.001) Differences in diabetes distress, depression, diabetes symptoms, blood pressure and lipid levels were not observed.
Yoo HJ, 2009[20]	Intervention group: n = 57/57.0(9.1) Control group: n = 54/59.4(8.4)	Randomized controlled trials/3 months	Korea (Telemonitoring system)	The UCDC system (reminding the participant to measure their blood glucose, blood pressure and body weight through cellular phones)Through to remind the enter and body weight). The intervention group: Providing a cellular phone with a modular blood glucose measuring device, an automatic blood pressure monitoring device, and body weight scales.	The feedbackalgorithm sent messages of encouragement, reminders, and recommendations after each data upload.	The intervention group: A significant improvements in HbA1c (7.6±0.9%to 7.1 ± 0.8%, P < 0.001), SBP, DBP, total cholesterol, LDL-c and triglyceride levels. The control group: A significant improvements in HbA1c (7.4±0.9% to 7.6 ± 1.0%, P = 0.03) Hs CRP and interleukin-6 levels did not change in either group.
Marı′a I RI, 2009[21]	Intervention group: n = 161/63.32 (61.60, 65.04) Control group: n = 167/64.52(62.96, 66.09)	Randomized controlled parallel-group trial/1y	Spain (Telemonitoring system)	The teleassistance system, DIABECOM: An ACCU-Chek Compact glucometer,a mobile phone and the call center. The intervention group: Providing a mobile phone with a teleassistance system (real-time transmission of blood glucose results and telephone consultations).	Physicians could contact their patients via mobile phones based on the information patients uploaded.	The intervention group: A reduction in HbA1c from 7.62±1.60% to 7.40±1.43% (P = 0.027) and a significant decrease in SBP, DBP, total cholesterol, LDL-c, and BMI. The control group: A reduction in HbA1c from 7.44±1.31% to 7.35±1.38% (P = 0.303) and a significant decline in LDL-c.
Quinn CC, 2008[22]	Intervention group: n = 13 Control group: n = 13 Overall mean age: 51.04(11.03)	Randomized controlled trials/3 months	U.S. (Mobile phones)	“WellDoc^™^”: The software provided real-time feedback on patients’ blood glucose levels, displayed patients’ medication regimens, with hypo- and hyperglycemia treatment algorithms. The intervention group: Providing a mobile phone with “WellDoc^™^”	The feedback information about the patient-specific target level and HCP(health care provider)—prescribed medication instructions was given once the blood glucose was labeled.	The intervention group: The average decrease in HbA1c was 2.03% and 84% had medications changed by HCP compared to controls (23%, P = 0.002). The control group: The average decrease in HbA1c was 0.68%
Cho JH, 2009[23]	Phonegroup: n = 35/51.1(13.2) Internet group: n = 34/45.2(11.3)	A randomized trial/3 months	Korea (Telemonitoring system)	The telecommunication-based glucose control system: a mobile phone with the capacity to measure blood glucose. The intervention group: Providing the telecommunication system which patients could log on and uploaded their glucose data at any time.	The phone group: Reciving feedback through the mobile phone only. Internet group: Reciving medical recommendations by short message service.	The Internet group: HbA1c levels decreased significantly from 7.6% to 6.9% as well as two-hour postprandial glucose level. The phone group: HbA1c levels decreased significantly from 8.3% to 7.1% as well as two-hour postprandial glucose level. Fasting plasma glucose levels did not change and levels of patient satisfaction were similar between groups.
Istepanian RS, 2009[24]	Intervention group: n = 66/60(12) Control group: n = 60/57(13)	Randomized controlled trials/9 months(mean)	U.K. (Mobile phones)	A mobile phone with a bluetooth wireless link to a blood glucose sensor. The intervention group: Patients were given the mobile phone and trained to measure the blood glucose.	Clinicians examined and responded to the data uploaded as a feedback.	No significant difference in HbA1c between the intervention and control groups was observed. Sub-group analysis: A lower HbA1c in telemonitoring group than control group: 7.76% and 8.40%, respectively (P = 0.06).
Seto E, 2009[25]	UK study: same as article 10; Canadian studies: The GTA study: n = 33/58.1(9.9); The Chapleau study: n = 26/63.7(8.7)	Canadian studies: Randomized controlled trials/4 months	Canadian (Telemonitoring system)	The diabetes and hypertension telemonitoring system: Mobile phone, glucometer and web server. The intervention group: The GTA study: Only blood pressure were monitored using Blood Pressure Monitor; The Chapleau study: monitor Both blood glucose and blood pressure were monitored.	Automated voice messages were sent to the patients after data uploading.	The GTA study: The average SBP dropped 9 mmHg and DBP dropped 3 mmHg (p<0.001/0.005 systolic/diastolic). The Chapleau study: No significant decrease in blood pressure was observed.
van der Weegen S, 2015[26]	Group 1: 65/57.5(7.0) Group 2: 66/56.9(8.3) Group 3: 68/59.2(7.5)	Randomized controlled trials/4-6 months	Netherlands (Mobile phones)	The It’s LiFe! system: Behavior change strategies and a monitoring and feedback tool. 3-arm: group 1: received self-management support program (SSP) combined with the monitoring and feedback tool; group 2: the SSP; group 3: care as usual	The feedback tool: A technology inside mobile phones in combination with pedometers or accelerometers which could answer questions via a dialogue session on the tool.	More physical activity directly was showed in Group 1 than Group 3 (mean difference 11.73, 95% CI 6.21–17.25; P<0.001), and Group 2 (mean difference 7.86, 95% CI 2.18–13.54;P = 0.003) after the intervention.
Quinn CC, 2014[27]	Intervention group: n = 62/52.0(8.0) Control group: n = 55/53.3(8.4)	Randomized controlled trials/1 year	U.S. (Mobile phones)	The coaching system:A mobile phone application to record information about diabetes self-management(blood glucose levels, carbohydrates consumed, diabetes medications taken) Main study measures: Medication records (medication, dose, frequency, duration) at baseline and study end.	An automatic real-time and personalized coaching feedback.	The intervention group: A higher percentage of patients had modification and intensification of incretin mimetics (9.7% vs 0.0% and 8.1% vs 0.0%). Physician prescribing of oral antihyperglycemic medications: No statistically significant difference was observed.
Hsu WC, 2016[28]	Intervention group: n = 20/53.3 Control group: n = 20/53.8	Randomized controlled trials/12±2 weeks	U.S. (Telemonitoring system)	The diabetes management program: Selftracking tools, decision-making interfaces and streamlined communications tools (secure text messages and virtual visits). The intervention group: Providing care through the cloud-based diabetes management program.	Shared decision-making interfaces and secure text messages.	A greater HbA1c decline and more treatment satisfaction in the intervention group compared with the control group.

### Data synthesis and analyses

Thirteen studies were selected for the systematic review, of which six with both primary and secondary outcomes containing 1,022 patients were included for the meta-analysis [16–21]. The sample size, the mean change of HbA1c[16–19, 21] or the mean ± standard deviation (SD) value of HbA1c at baseline and the endpoint[20] were extracted. The other seven studies had several data points unavailable; for example, the mean changes in HbA1c levels or the SD or 95% confidence interval (95% CI) of HbA1c were missing[22–24], or had only descriptive results reflecting changes in blood glucose, life-style behaviors, and medicine after using the apps[25–28]. Extracted data of thirteen studies are presented in Table 1.

In the meta-analysis, data from the intervention groups were combined and compared to the control groups. The data were obtained from original studies selected or calculated[29] from the raw data; the value for standard deviation (SD) was frequently estimated using the reported 95% CI and p-values. We also performed a subgroup analysis to assess whether change in HbA1c differed based on average HbA1c at baseline. A random-effects model was used for pooling the included studies, as clinical heterogeneity was expected. Heterogeneity was identified by visual inspection of the forest plots and statistically examined with the inconsistency I^2^ test, in which values greater than 50% were considered highly heterogeneous. A sensitivity analysis was performed only for the primary outcome; in the sensitivity analysis, z-test was used for hypothesis testing of the overall pooled effect. A p-value <0.05 was considered statistically significant.

Statistical analysis was performed using Review Manager 5.3.

## Results

### Descriptions of Studies

**Fig 1:**
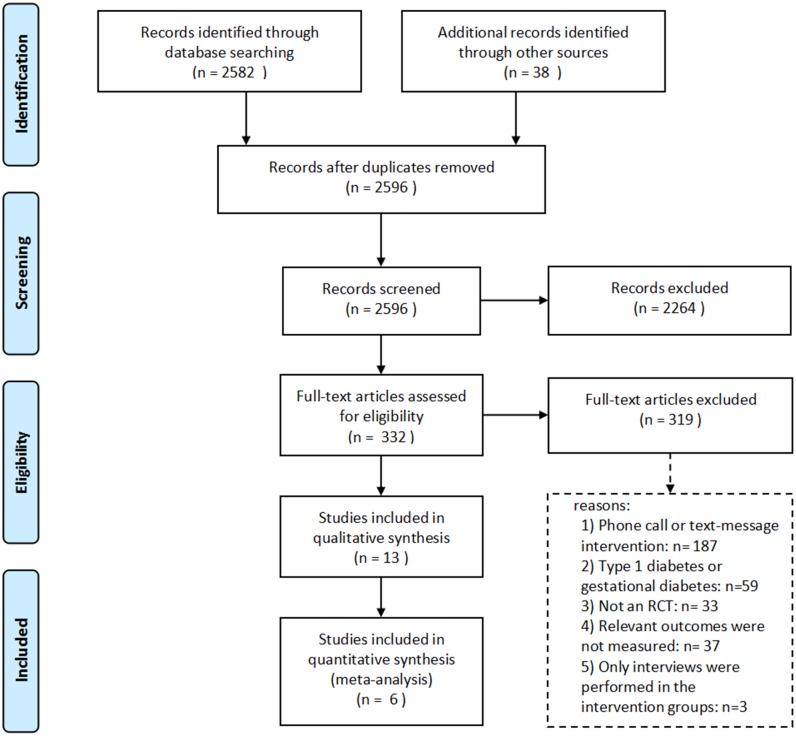
Flow diagram for the scientific paper selection from databases.

Literature review resulted in 2,596 articles after excluding duplications, including 739 from PubMed, 961 from EMBASE database, 882 from the Cochrane Library and 14 from references of articles identified through database. Only 13 articles fulfilled our selection criteria. Fig 1 shows the flow diagram for the paper selection from the three databases. Additional information, including the name and description of the mobile apps in each study, their modules and function, the feedback system of each app, the length of follow-up, sample size, mean age, aim, and summary of the study results, was extracted and is shown in Table 1.

This meta-analysis contained 1,022 subjects merged from six studies, among which, 58% were men. There were 533 people in the mHealth-intervention group, 74 in the mHealth-no feedback group, and 415 in the usual care group. The numbers of subjects in each individual study ranged from 24[18] to 180[16]. All study participants had type 2 diabetes. In one study[16], the participants had both type 2 diabetes and heart disease. All studies except one[16] reported a mean duration of diabetes ranging between 3 and 12 months. The duration of the intervention was less than 6 months in 6 studies and more than 6 months in 7 studies. Five studies took place in the United States, 2 in Canada, 2 in Korea and 1 each in Spain, Norway, Netherlands, Finland and the United Kingdom. The mean age of participants ranged from 45.2[23] to 66.6[16] years old.

**Fig 2:**
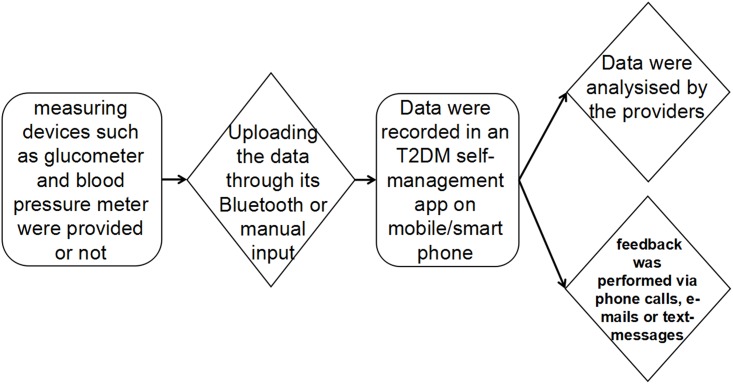
A model to demonstrate how self-management apps work.

Among the 6 studies included in the meta-analysis, one[17] that performed a three-arm study that included two intervention groups and one control group, and another performed a four-arm study[19] that included three intervention groups and one control group. All six studies of the meta-analysis included personalized feedback from a healthcare practitioner to the patient or other healthcare provider regarding the clinical data uploaded. Five[16–19, 21] of all the trials provided information about the frequency of feedback of information to patients using the form of phone calls ranging from once a week to once a month. One[20] study incorporated a feedback algorithm developed by endocrinologists via messages. Although the strategies of mHealth apps is diverse, all of them comprise four parts including a mobile/smartphone with self-management apps, measuring devices, patients who upload the data to the apps and providers who analysis the data and perform the feedback. So the principle and flow paths of mHealth were similar (Fig 2).

### Overall Study Quality and Publication Bias Assessment

Among all the studies selected for meta-analysis, five presented random sequence generation, three reported allocation concealment, blinding of participants and personnel and blinding of outcome assessment occurred in one. All six studies reported the incomplete outcome data. Details of the risk of bias assessment were showed in S3 Table.

### Study Outcomes

#### Main outcomes

**Fig 3:**
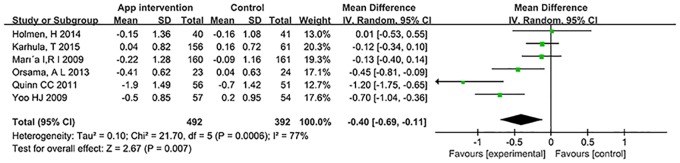
Forest plot for HbA1c level in studies with feedback group.

We found that mobile phone app strategies were associated with a significant reduction in HbA1c by -0.40% (-4.37 mmol/mol) (95% CI -0.69 to -0.11% [-7.54 to -1.20 mmol/mol]; p = 0.007) in mean difference (MD) (Fig 3) and -0.40% (-4.37 mmol/mol) (95% confidence interval [CI] -0.69 to -0.10% [-7.54 to -1.09 mmol/mol]; p = 0.008) in standardized mean differences (SMD) when compared to standard diabetes care. However, there was substantial heterogeneity in the overall pooled effect (I^2^ = 77%).

Other three studies with main outcomes[22–24] indicated a clinically significant mean HbA1c reduction in the intervention group and the study by J.H. Cho[23] found that the mean HbA1c levels decreased significantly (p<0.01) in both the phone group and the internet group, and the decrement between the two groups was not significantly different (P = 0.27).

Four studies[16, 18–20] assessed the effect of mHealth on SBP and DBP. There was no significant effect of mHealth apps strategies on SBP -2.62 mmHg; 95% CI -5.60 to 0.36 mmHg; p = 0.08) or DBP (effect size -1.76 mmHg; 95% CI -3.6 to 0.07 mmHg; p = 0.06) when compared to usual care. There was no evidence of heterogeneity in the studies evaluating SBP or DBP (SBP I^2^ = 0%, p for heterogeneity = 0.95; DBP I^2^ = 0%, p for heterogeneity = 0.76) (Fig A and Fig B in S1 File).

Three studies[16, 19, 20] measuring serum lipids were combined in the meta-analysis. There was no significant difference between the intervention group and control group (effect size: -0.12 mmol/l, 95% CI -0.34 to 0.11 mmol/l, p = 0.30; 0.01mmol/l, 95% CI -0.05 to 0.07 mmol/l, p = 0.81;-0.06mmol/l, 95% CI -0.32 to 0.19 mmol/l, p = 0.62; -0.15 mmol/l, 95% CI -0.6 to 0.3 mmol/l, p = 0.50 for LDL-c, HDL-c, TG and TC, respectively). There was no evidence of heterogeneity for HDL-c and TG (HDL-c I^2^ = 0%, p for heterogeneity = 0.69; TG I^2^ = 0%, p for heterogeneity = 0.62). However, there was substantial heterogeneity for TC (I^2^ = 77%, p for heterogeneity = 0.01) and moderate heterogeneity for LDL-c (I^2^ = 43%, p for heterogeneity = 0.18) (FigC, FigD, FigE and FigF in S1 File).

Four studies assessed the effect of mHealth on weight[16–18, 20]. However, these studies demonstrated no significant reduction in weight for those using mHealth compared to those not using mHealth (effect size: -0.84 kg, 95% CI: -2.04, 0.36 mmol/l, p = 0.17), and there was low heterogeneity (I^2^ = 30%, p for heterogeneity = 0.23) (Fig G in S1 File).

There were two studies that looked at changes in blood glucose. One study found significantly decreased two-hour postprandial glucose levels in both the phone group and the internet group after three months[23]; however, fasting plasma glucose were not significantly different in either study[20].

#### Behavioral outcomes

There was only 1 article[26] that explored the effects of mHealth interventions on physical activity. The main outcome measure was minutes of physical activity per day. In the group that received app-directed self-management combined with the monitoring and feedback tool, there was significantly more physical activity after three-month follow-up compared to the mHealth group that did not receive feedback. The mean difference in physical activity per day was 10.59 minutes (95% CI: 4.94, 16.25; P<0.001). Compared to the usual-care group, the mean difference was 9.41 minutes (95% CI 3.70, 15.11; P<0.001).

#### Medicine use changes

There were two studies[27, 28] that aimed to test whether the medication usage was affected by engaging in an mHealth app. However, the overall difference in physician prescribing of oral antihyperglycemic medications was not statistically significant. Of note, patients that started basal insulin achieved better glycemic control compared with standard clinical practice.

#### Subgroup analyses

As there were two studies with more than two treatment arms, including both intervention groups with and without feedback, we did a subgroup analysis to see if there was a difference for the combined effect between the two methods. When outcomes for “mHealth with feedback” were combined, the overall effect size for HbA1c reduction was statistically significant: -0.40% (-4.37 mmol/mol) (95% CI -0.69 to -0.11% [-7.54 to -1.20 mmol/mol]; p = 0.008) with substantial heterogeneity (I^2^ = 77%). When outcomes of studies were combined that measured effects of “mHealth without feedback,” the overall effect size for HbA1c reduction was larger, but no longer statistically significant: -0.46% (-5.03 mmol/mol) (95% CI -1.19 to 0.26% [-13.0 to 2.84 mmol/mol]; p = 0.21) with similar heterogeneity of 62%.

As the interventions showed significant heterogeneity in the overall pooled result (I^2^ = 77%), a sensitivity analysis was done to explore possible reasons. An analysis was performed excluding two studies for methodological reasons. One study[20] noted that the intervention duration was only 3 months, which was much shorter than other studies. The other study[19] used cluster randomization but analyzed results as if they were from individually randomized trials. Additionally, there were specific feedback algorithms used to send educational and motivational messages to patients after each data upload in the two studies instead of factitious feedback methods adopted in others. Removing these two studies decreased the pooled effect to -0.17% (-1.86 mmol/mol) (95% CI -0.32 to -0.02% [-3.50 to -0.22 mmol/mol]; p = 0.03) and decreased heterogeneity to 2% while after removing one of these two studies, the heterogeneity did not decreased obviously.

**Fig 4:**
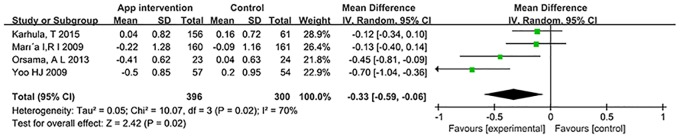
Forest plot for HbA1c < 8% at baseline levels.

According to the American Diabetes Association (ADA), there are multiple factors that dictate the target HbA1c for individuals living with diabetes mellitus; for patients with more severe disease, control of diabetes to achieve an HbA1c of 8% (64.0 mmol/mol) is recommended [30]. We had divided the studies based on the baseline HbA1c into two subgroups (>8% and <8%) using data presented in the feedback strategy of all selected studies (S2 Table). When the studies were combined for patients with HbA1c > 8% at baseline, there was no significant difference between the intervention group and control group (p = 0.33). However, when data from the subgroup of individuals with baseline HbA1c < 8% was reviewed, the overall effect size for HbA1c in MD was statistically significant: -0.33% (-3.61 mmol/mol) (95% CI: -0.59, -0.06% [-6.45, -0.66 mmol/mol]; p = 0.02) with a heterogeneity of 70% (Fig 4) and -0.38% (-4.15 mmol/mol) (95% CI -0.71 to -0.05% [-7.76 to -0.55 mmol/mol]; p = 0.02) in SMD with a heterogeneity of 74%. When one study[20] was removed for methodological reasons (as mentioned above), the pooled effect of MD decreased to -0.19% (-2.08 mmol/mol) (95% CI: -0.36, -0.02% [-3.93, -0.22 mmol/mol]; p = 0.03) with a decreased heterogeneity of 23%.

## Discussion

We performed a systematic review and meta-analysis to assess the effect of mHealth apps on glycemic self-control in T2DM patients. Mobile-phone/smartphone-based self-management apps appear to have moderate benefits on glycemic control with a pooled effect on HbA1c reduction of -0.40% (-4.37 mmol/mol), indicating that the mHealth app intervention could improve T2DM patients’ glycemic conditions. The result is in accordance with some previous systematic reviews regarding self-management of diabetes[20]. One study showed that the pooled effect on HbA1c reduction was -0.2% after computer-based diabetes self-management interventions[11], and another study showed a decrease in HbA1c of -0.44% after a telemedicine intervention for the care of diabetes[31].

As the first meta-analysis comparing the difference between effects from app with and without feedback, the overall effects on HbA1c reduction were no longer statistically significant for those who received no feedback. This evidence may suggest that mHealth app creators should incorporate a feedback module into the design of the application. Additionally, patients with milder disease with baseline level of HbA1c lower than 8% appeared to benefit more from mHealth apps compared with those with higher baseline HbA1c. This result may be counterintuitive and implies that patients with better plasma glucose control at baseline may have a better response to an mHealth app intervention. Patients with lower HbA1c levels is easier to be controlled under interventions[32].

We did not find significant differences in SBP and DBP post-intervention. These results are similar with other previous studies that showed no significant differences in SBP or DBP after telemedicine intervention[31, 33]. But our result showed that the p-value of the change in SBP as well as DBP in mHealth invention group compared with control group was close to 0.05. Maybe the small sample size of this meta-analysis resulted in the negative results and further studies using mHealth to assist controlling blood pressure will contribute to clarify it. Furthermore, only few studies used blood glucose levels as an outcome; therefore data was too limited to perform an analysis about the blood glucose fluctuations, which cannot be assessed from HbA1c levels alone.

Currently available mHealth interventions also appeared to be moderately effective in promoting lifestyle changes, including daily physical activity and changes in medicine requirements. Mobile health apps may have a benefit for those attempting to adopt good habits in daily life. However, a meta-analysis conducted last year suggested that mobile phone app interventions reduced body weight by only 1.04 kg, reduced BMI by only 0.43 kg/m^2^, and nonsignificantly increased physical activity by a standardized mean difference of 0.40 compared with various control interventions [34]. The discrepancy between this study and our results may be explained by the inclusion of participants without disease except obesity as well as the various control interventions that were included in their study. Moreover, the current review showed that patients starting basal insulin achieved better glycemic control compared with standard clinical practice. However, the overall difference in physician prescribing of oral antihyperglycemic medications was not statistically significant.

There exist several systematic reviews analyzing the difference between Internet-based, computer-based and telephone-based interventions and standard of care only in self-management of diabetes. Some previous reviews looked at the effect of mobile phone-based interventions on diabetes self-management [33] and showed the potential power of mHealth in diabetes care. There exist different types of smartphone applications regarding T2DM self-management, such as “Easy Diabetes”, “Diabetes Manager”, “myDiabetes” and so on[35]. Numerous characteristics of these apps including increased accessibility to educational resources and self-management strategies, more frequent physical and emotional symptom tracking, and increased access to peer support are all main strengths of them in benefiting self-management of individuls[36, 37]. However, there was no previous meta-analysis evaluating the impact of mHealth apps on glycemic control in T2DM patients. With the continual development of better handheld technology, mHealth apps may increasingly benefit patients with T2DM to care for themselves.

Considering the application of new and advanced technology to clinical practice, increasing patients’ awareness of the potential consequences of severe disease without good self-management and of the various kinds of smartphone apps with potential health benefits are important. Fortunately, patients have expressed high interest as well as satisfaction in smartphone apps in preliminary studies[38–40].

Although only 1 of 6 studies in this meta-analysis had blinded assessment of outcomes, a low risk of bias was found among them. However, the substantial heterogeneity between the studies was the main limitation of this meta-analysis. We have performed analyses to identify methodological reasons of studies. As the analysis was not based on individual data, the subgroup analysis comparing studies with a mean HbA1c level lower or higher than 8.0% (64.0 mmol/mol) was limited. We did not have sufficient information to separate individual patients with better and worse glycemic control. However, our study presented an overall rough estimate that provides an area for further research.

## Conclusions

This systematic review and meta-analysis has shown that diabetes self-management with mHealth apps may help to manage T2DM and have a moderate beneficial effect on glycemic control. Although there was no clinical relevant impact on blood pressure, serum lipids, or weight, the effect of these outcomes should be further explored in future trials. More applicable mHealth apps should be designed, and more rigorous studies are needed to further explore aspects of diabetes self-management that can be brought into clinical practice.

## Supporting Information

S1 File Results of secondary outcomes. (DOCX)

S1 PRISMA Checklist PRISMA 2009 checklist. (DOC)

S1 Table Search strategy for PubMed. (DOCX)

S2 Table Baseline of HbA1c. (DOCX)

S3 Table Risk of bias assessment. (DOCX)
