# Supplementary material for: T2DM Self-Management via Smartphone Applications: A Systematic Review and Meta-Analysis
Source: PLoS One. 2016 Nov 18;11(11):e0166718. doi: 10.1371/journal.pone.0166718 (PMC5115794; doi:10.1371/journal.pone.0166718)
Supplement: S1 File — (DOCX) [file pone.0166718.s001.docx]

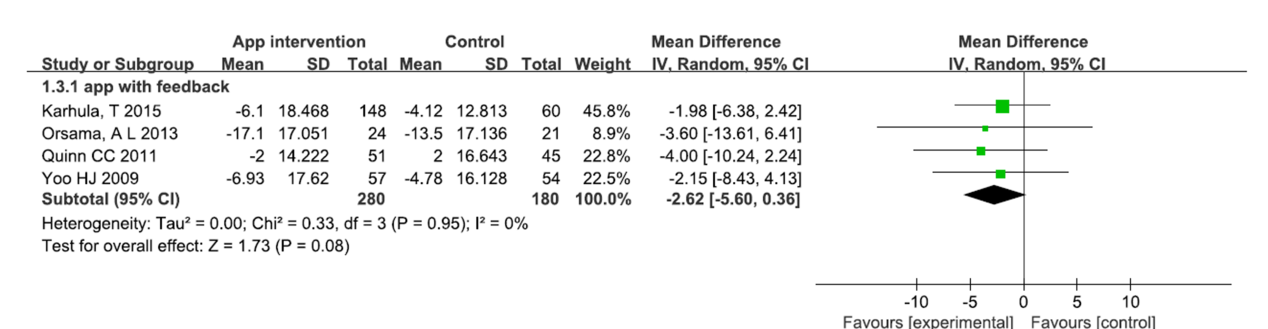


Fig A. Forest plots for SBP (mmHg).


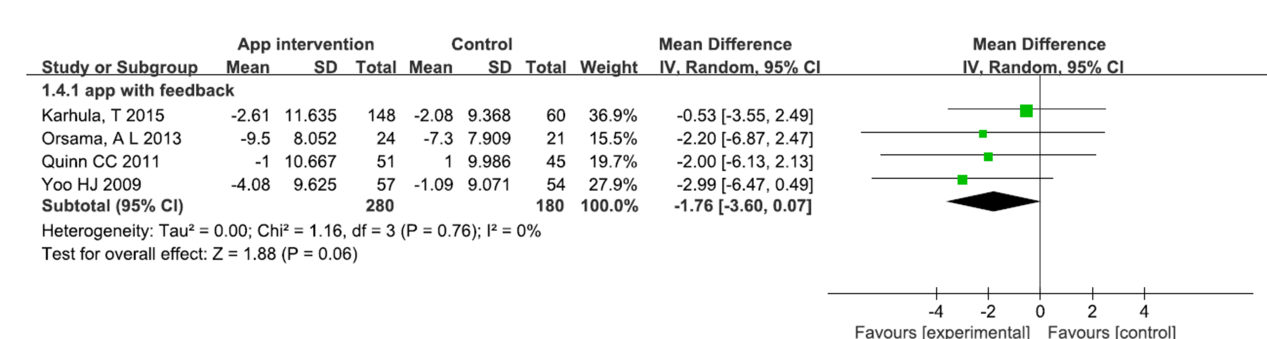


Fig B. Forest plots for DBP (mmHg).


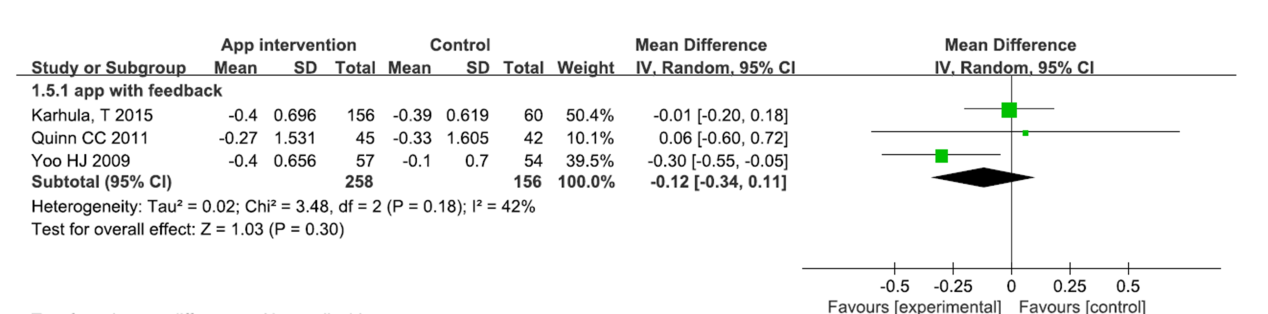


Fig C. Forest plots for LDL-c (mmol/l).


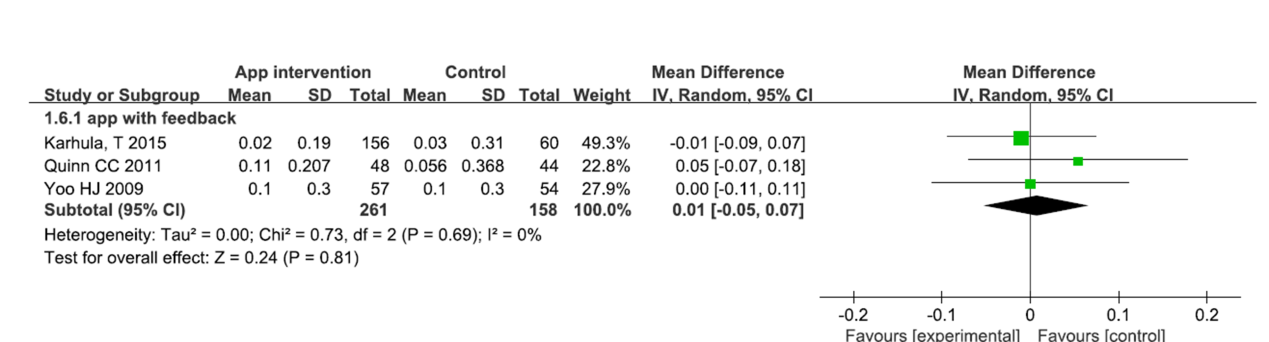


Fig D. Forest plots for HDL-c (mmol/l).


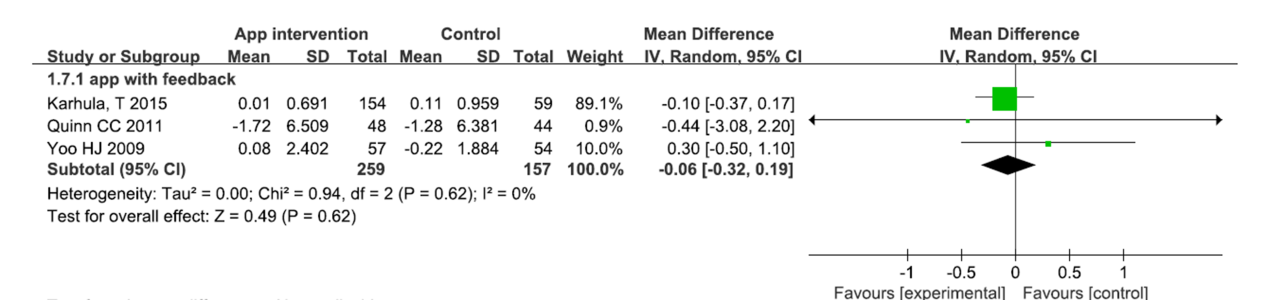


Fig E. Forest plots for TG (mmol/l).


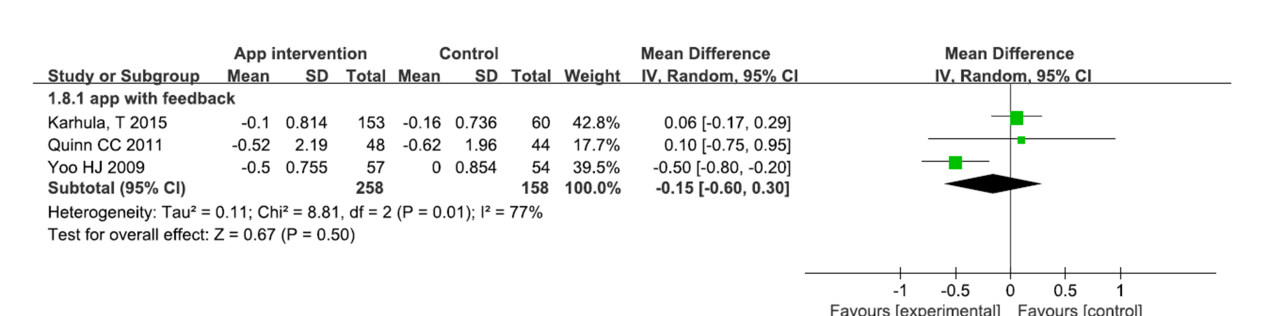


Fig F. Forest plots for TC (mmol/l).


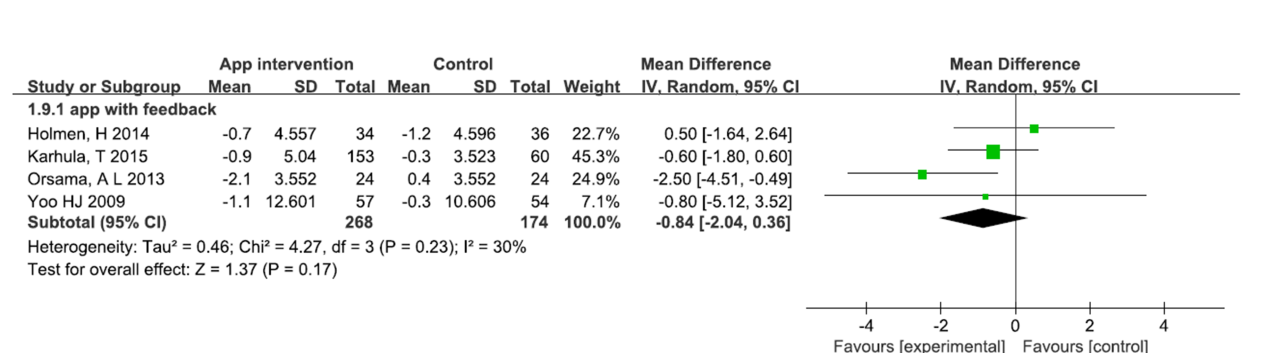


Fig G. Forest plots for weight (kilogram).
