# Supplementary material for: T2DM Self-Management via Smartphone Applications: A Systematic Review and Meta-Analysis
Source: PLoS One. 2016 Nov 18;11(11):e0166718. doi: 10.1371/journal.pone.0166718 (PMC5115794; doi:10.1371/journal.pone.0166718)
Supplement: S1 Table — (DOCX) [file pone.0166718.s003.docx]

S1 Table.Search strategy for PubMed.

| #1 | Search "Diabetes Mellitus, Type 2"[Mesh] | 95523 |
| --- | --- | --- |
| #2 | Search type 2 diabetes mellitus | 105044 |
| #3 | Search (NIDDM or T2DM or T2D) | 108368 |
| #4 | Search (non insulin* depend* or noninsulin* depend* or non insulin?depend* or noninsulin?depend*) | 12207 |
| #5 | Search (insulin* depend* or insulin?depend*) | 67996 |
| #6 | Search (#1 OR #2 OR #3 OR #4 OR #5) | 163419 |
| #7 | Search "Self Care"[Mesh] | 43379 |
| #8 | Search Glycemic Control | 19339 |
| #9 | Search glucose control | 111008 |
| #10 | Search self-management | 158243 |
| #11 | Search self-care activities | 12540 |
| #12 | Search self-monitoring | 8563 |
| #13 | Search blood glucose monitoring | 13556 |
| #14 | Search (#7 OR #8 OR 9 OR #10 OR #11 OR #12 OR #13) | 277243 |
| #15 | Search "Telemedicine"[Mesh] | 17837 |
| #16 | Search "Cell Phones"[Mesh] | 6645 |
| #17 | Search "Smartphone"[Mesh] | 131 |
| #18 | Search "Self-Evaluation Programs"[Mesh] OR "Programs" [Publication Type] OR "Managed Care Programs"[Mesh] OR "Mobile Applications"[Mesh] OR "Software"[Mesh] | 160076 |
| #19 | Search (((((cellular phone* or cell phone* or mobile phone* or telephone*)) OR (mobile health or telehealth or telehealthcare or electronic health*)) OR health information technology) OR phone intervention) OR app* | 990632 |
| #20 | Search (#15 OR #16 OR #17 OR #18 OR #19) | 1097277 |
| #21 | Search randomized controlled trial [pt] | 405976 |
| #22 | Search controlled clinical trial [pt] | 491181 |
| #23 | Search randomized [tiab] | 369269 |
| #24 | Search placebo [tiab] | 173385 |
| #25 | Search drug therapy [sh] | 1816391 |
| #26 | Search randomly [tiab] | 248078 |
| #27 | Search trial [tiab] | 420771 |
| #28 | Search groups [tiab] | 1574177 |
| #29 | Search (#21 OR #22 OR #23 OR #24 OR #25 OR #26 OR #27 OR #28) | 3750853 |
| #30 | Search (animals [mh] NOT humans [mh]) | 4181678 |
| #31 | Search (#29 NOT #30) | 3235146 |
| #32 | Search (#6 AND #14 AND #20 AND #31) | 1009 |
