# Supplementary material for: T2DM Self-Management via Smartphone Applications: A Systematic Review and Meta-Analysis
Source: PLoS One. 2016 Nov 18;11(11):e0166718. doi: 10.1371/journal.pone.0166718 (PMC5115794; doi:10.1371/journal.pone.0166718)
Supplement: S2 Table — (DOCX) [file pone.0166718.s004.docx]

S2 Table. Baseline of HbA1c.

| Author, year | Group (sample size) | Glycated hemoglobin (SD or 95%CI) (%) |
| --- | --- | --- |
| Karhula T, 2015* | Intervention group(n=180) | 7.25 |
|  | Control group(n=70) | 7.2 |
| Holmen H, 2014 | FTA (n=51) | 8.1 (1.1) |
|  | FTA-HC (n=50) | 8.2 (1.1) |
|  | Control group (n=50) | 8.3 (1.2) |
| Orsama AL, 2013 | Intervention group (n = 24) | 6.86 (1.56) |
|  | Control group (n = 24) | 7.09 (1.51) |
| Quinn CC, 2011 | Group 1: UC (n = 56) | 9.2 (1.7) |
|  | Group 2: CO (n = 23) | 9.3 (1.8) |
|  | Group 3: CPP (n = 22) | 9.0 (1.8) |
|  | Group 4: CPDS (n = 62) | 9.9 (2.1) |
| Yoo HJ, 2009 | Intervention group (n = 57) | 7.6 (0.9) |
|  | Control group (n = 54) | 7.4 (0.9) |
| Marı´a I RI, 2009 | Telemedicine group(n=161) | 7.62 (7.38, 7.88) |
|  | Control group(n=167) | 7.41 (7.21, 7.61) |

* There is no SD or 95%CI available in the study.
