# Supplementary material for: T2DM Self-Management via Smartphone Applications: A Systematic Review and Meta-Analysis
Source: PLoS One. 2016 Nov 18;11(11):e0166718. doi: 10.1371/journal.pone.0166718 (PMC5115794; doi:10.1371/journal.pone.0166718)
Supplement: S3 Table — (DOCX) [file pone.0166718.s005.docx]

S3 Table. Risk of bias assessment.

| study | Random sequence generation | Allocation concealment | Blinding of participant and personnel | Blinding of outcome assessment | Incomplete outcome data | Selective reporting |
| --- | --- | --- | --- | --- | --- | --- |
| Karhula T 2015 | Yes | Yes | Yes | Unclear | Yes | Unclear |
| Holmen H 2014 | Yes | No | No | Unclear | Yes | Unclear |
| Orsama AL 2013 | Yes | Unclear | Unclear | Unclear | Yes | Unclear |
| Quinn CC 2011 | Yes | Yes | Unclear | Yes | Yes | Unclear |
| Yoo HJ 2009 | Unclear | Unclear | Unclear | Unclear | Yes | Unclear |
| Marı´a I RI 2009 | Yes | Yes | No | No | Yes | Unclear |
